# Acceptance and commitment therapy reduces psychological distress in patients with cancer: a systematic review and meta-analysis of randomized controlled trials

**DOI:** 10.3389/fpsyg.2023.1253266

**Published:** 2024-01-05

**Authors:** Xing Jiang, Jian Sun, Ruiwen Song, Yue Wang, Jinglian Li, Rongwei Shi

**Affiliations:** ^1^School of Nursing, Nanjing University of Chinese Medicine, Nanjing, China; ^2^Department of General Surgery, Jiangsu Province Hospital of Chinese Medicine, Affiliated Hospital of Nanjing University of Chinese Medicine, Nanjing, China; ^3^Department of Internal Medicine, Jiangsu Province Hospital of Chinese Medicine, Affiliated Hospital of Nanjing University of Chinese Medicine, Nanjing, China

**Keywords:** acceptance and commitment therapy, neoplasms, cancer, psychological distress, anxiety, systematic review, meta-analysis

## Abstract

**Objective:**

This study aimed to systematically review and meta-analyze the clinical efficacy of acceptance and commitment therapy (ACT) in patients with cancer and psychological distress.

**Methods:**

Randomized controlled trials (RCTs) from seven English electronic databases were systematically investigated from inception to 3 October 2023. A total of 16 RCTs from 6 countries with 711 participants were included in this study. Estimated pooled effect sizes (ESs) were calculated via inverse-variance random-effects or fixed-effects (I^2^ ≤ 50%) model and presented by standardized mean difference (SMD). Subgroup analyses were performed to reduce confounding factors and heterogeneity, and the Grading of Recommendations Assessment, Development, and Evaluation (GRADE) system was used to evaluate the quality of the pooled ESs.

**Results:**

The pooled ESs revealed that statistically significant improvements in anxiety [postintervention SMD = −0.41 (95% confidence interval (CI), −0.71, −0.11); *p* = 0.008; I^2^ = 65%; follow-up SMD = −0.37 (95% CI, −0.66, −0.08); *p* = 0.01; I^2^ = 29%], depression [postintervention SMD = −0.45 (95% CI, −0.63, −0.27); *p* < 0.001; I^2^ = 49%; follow-up SMD = −0.52 (95% CI, −0.77, −0.28); *p* < 0.001; I^2^ = 0%], and psychological flexibility [postintervention SMD = −0.81 (95% CI, −1.50, −0.11); *p* = 0.02; I^2^ = 84%; follow-up SMD = −0.71 (95% CI, −1.12, −0.31); *p* = 0.0006; I^2^ = 38%] in ACT-treated participants were observed compared to patients treated with control conditions. However, other outcomes, such as physical symptom alleviation, were not significantly associated.

**Conclusion:**

The findings of this systematic review and meta-analysis suggest that ACT is associated with improvements in anxiety, depression, and psychological flexibility in patients with cancer.

**Systematic review registration:**

https://www.crd.york.ac.uk/prospero/display_record.php?ID=CRD42022320515.

## 1 Introduction

Cognitive-behavioral therapy (CBT) has been widely recognized as the primary psychotherapy intervention for addressing various mental disorders, including anxiety, depression, and schizophrenia (Andersson et al., 2019; Cuijpers et al., 2019). However, the initial two generations of CBT, specifically traditional behavior therapy and cognitive-behavioral therapy, have demonstrated limitations such as the poor link between existing clinical traditions and basic principles, vague definitions of interventions, and weak evidence to support the efficacy of these interventions, which makes therapies relatively mechanistic and uncertain (Hayes, 2016). Based on the first and second waves of CBT, the third wave, including acceptance and commitment therapy (ACT), mindfulness-based cognitive therapy (MBCT), dialectical behavior therapy (DBT), and metacognitive approaches, seems to carry the CBT tradition forward into new territory. Instead of directly changing control behavior and cognition and suppressing or eliminating specific psychological issues, they prefer structuring flexible and effective repertoires such as mindfulness, acceptance, or cognitive defusion to alter the function of the individual’s relationship with these problems. ACT has stood out as the most representative and practical psychotherapy in recent years because of its theoretical foundation in relational frame theory (RFT) and the pragmatic philosophy of functional contextualism (Hayes et al., 2006). Instead of being committed to counterproductive attempts to control or eliminate undesirable thoughts, feelings, and experiences such as pain, anxiety, or fear, ACT primarily aims to develop greater psychological flexibility to help individuals productively adapt to these challenges with an influenced relationship with cognition through six core processes. The six processes are acceptance, cognitive defusion, being present, the self as context, values, and committed action (Hofmann and Asmundson, 2008; Hayes et al., 2013; Dindo et al., 2017). Cancer diagnosis and treatment constitute profoundly stressful experiences, such as anxiety and depression, which influence cancer progression (Dai et al., 2020; Mravec et al., 2020; Eckerling et al., 2021), negatively affect quality of life (QoL), and are closely associated with cancer-specific and all-cause mortality (Batty et al., 2017; Wang Y. H. et al., 2020). There is a reciprocal causal relationship between psychological distress and symptoms such as fatigue (Bower et al., 2014; Berger et al., 2015), functional limitations, pain, sleep problems, and sadness (Mehnert et al., 2018; Antoni et al., 2023), and situations such as financial toxicity (Ramsey et al., 2016; Altice et al., 2017; Carrera et al., 2018) and post-traumatic stress disorder (PTSD) (Cordova et al., 2017; Wang Y. Y. et al., 2020). In the case of patients with cancer, ACT applies acceptance and mindfulness processes and value-based living and commitment processes to generate psychological flexibility where patients are guided to actively and unjudgmentally experience (not merely tolerate) the cancer conditions here and now as they are, explore and clarify values, identify achievable goals, and commit concrete actions to overcome the specific barriers hindering the steps toward the value ends (Hawkes et al., 2014; Hayes, 2016). Owing to its adaptability, the ACT has been widely applied in various fields (Biglan et al., 2008; Ost, 2008; Powers et al., 2009; Andersson et al., 2014; Ost, 2014; A-Tjak et al., 2015; Graham et al., 2016; Carlbring et al., 2018; Andersson et al., 2019; Gloster et al., 2020; Thompson et al., 2021), including chronic pain (Daly-Eichenhardt et al., 2016; Du et al., 2021; Trindade et al., 2021), distress (Prudenzi et al., 2021), anxiety (Kelson et al., 2019), depression (Brown et al., 2016), insomnia (Hertenstein et al., 2014; Vethe et al., 2018), diabetes (Sakamoto et al., 2022), obsessive-compulsive disorder (OCD) (Bluett et al., 2014), social phobia (Craske et al., 2014), substance abuse (Luoma et al., 2012; Heffner et al., 2020), and hearing problems (Molander et al., 2018). Furthermore, ACT is flexible and can be delivered in various formats, including 1-day group workshops, face-to-face, telephone, and Internet-based ACT, providing multiple options well suited to the recent COVID-19 pandemic situation (Andersson et al., 2014, 2019; Washburn et al., 2021).

Several systematic reviews on the application of ACT in patients with cancer have recently been published (Li H. et al., 2021; Li Z. H. et al., 2021; Zhao et al., 2021; Fang et al., 2023; Maunick et al., 2023; Zhang et al., 2023). These studies summarized the efficacy of ACT on psychological distress or other symptoms (fatigue and sleep disturbance) in people with cancer. This study will provide some different findings in this field with relatively new and sufficient RCT data from English electronic databases. Reasonable heterogeneity or subgroup analyses and explanations combined with the Grading of Recommendations Assessment, Development, and Evaluation (GRADE) system would be made and utilized to improve the reliability of findings (Schünemann et al., 2013). We anticipate that this study will provide a distinct perspective and theoretical basis for clinical practice in this field.

This systematic review and meta-analysis aimed to provide a reliable estimate of the efficacy of ACT on psychological distress in adults living with cancer by comprehensively comparing the intervention and control groups. This study (1) primarily aimed to evaluate whether ACT intervention is associated with greater improvement in anxiety and depression than different control conditions with subgroup analyses, and (2) additionally assessed other prognosis or ACT-related outcomes, such as QoL and alleviation of other symptoms. Finally, the quality of evidence was examined using the GRADE system (Schünemann et al., 2013) to ensure transparency and confidence in the results.

## 2 Method

This study was conducted in accordance with (1) the PRISMA 2020 statement, an updated guideline for reporting systematic reviews (Page et al., 2021); (2) the Cochrane Handbook for Systematic Reviews of Interventions (Nasser, 2020); and (3) the GRADE Handbook for grading the quality of evidence and strength of recommendations (Schünemann et al., 2013). Before conducting this study, it was registered with CRD42022320515, the International Perspective Register of Systematic Reviews (PROSPERO).

### 2.1 Data sources and search strategy

A systematic search was conducted in seven English electronic databases: PubMed, Embase, Cochrane Library, Web of Science, ClinicalTrials.gov, EBSCO, and Elsevier, from inception to 12 December 2021, with a second supplementary search conducted on 3 October 2023. Medical Subject Heading (MeSH) terms, entry words from PubMed, and hedges (search filters) from Hedges Project, funding from the National Library of Medicine, and sources from the Health Information Research Unit (HIRU) of McMaster University^1^ were utilized, mainly including “acceptance and commitment therapy,” “neoplasm,” “cancer,” “tumor,” “malignancy,” “benign,” and “randomized controlled trial.” Please refer to Appendix A of the Supplementary Material for specific retrieval methods from PubMed and other databases.

### 2.2 Inclusion and exclusion criteria

Studies meeting the following “PICOS” criteria were considered eligible for inclusion (Moher et al., 2009).

1. P (Population): Participants who were at least 18 years old, diagnosed with various types of cancer (e.g., leukemia, multiple myeloma, melanoma, breast cancer, lung cancer, and ovarian cancer), and were still receiving or had completed any oncological treatment such as surgery, chemotherapy, and/or radiotherapy.
2. I (Intervention): ACT grounded in the perspective of Hayes et al. with six core operational processes or three broad response styles of Open, Aware, and Engaged or other versions of ACT (e.g., ACT matrix) would be the eligible intervention. ACT combined with other cognitive-behavioral therapies (i.e., behavioral activation) was also considered qualified for this meta-analysis.
3. C (Control): The types of controls involving treatment as usual (TAU) control, active control (AC) (i.e., group meeting, standardized talking control), and wait-list control (WL), which means that patients in the group will not receive treatments similar to the intervention groups until the end of the trial.
4. O (Outcome): (1) The primary outcome was psychological distress, including anxiety and depression; (2) The secondary outcome was specific indications related to the ACT process, such as psychological flexibility (measured by acceptance and commitment questionnaire-2, AAQ-2), and other symptoms (i.e., pain, fatigue, and insomnia) related to patients’ QoL.
5. S (Study design): Randomized controlled trial.

### 2.3 Screening procedure

EndNote X9 (Clarivate Analytics (US) LLC) was used to manage the primary search results, and duplicates were identified and deleted. Subsequently, two independent reviewers (JS and JL) conducted an initial screening to determine whether the titles and abstracts of the candidate articles met the inclusion criteria for the investigated topic. Furthermore, regarding potentially relevant and full-text-accessible articles, we conducted a thorough review of each individual to determine their inclusion status. During these procedures, a third reviewer (RuS) was introduced to resolve any remaining discrepancies after discussions between the first two reviewers and their independent judgments until a unified opinion was reached. The corresponding kappa values between the first two reviewers were calculated at the end of the screening procedure.

### 2.4 Risk-of-bias evaluation

The Revised Cochrane Risk-of-Bias tool for randomized trials (RoB 2.0) was used to assess the risk of bias in the RCTs (Higgins et al., 2016; Sterne et al., 2019). Five mandatory domains (randomization process, deviations from intended interventions, missing outcome data, measurement of the outcome, and selection of the reported results), which were identified based on both empirical evidence and theoretical considerations, were structured in the tool. One of three proposed risk-of-bias verdicts, “low risk of bias,” “some concerns,” or “high risk of bias,” would be reached via the specific algorithms according to the responses to signaling questions (i.e., yes, probably yes, probably no, no, and no information) for each domain. Then, users should conduct their verification and make changes when it is considered appropriate or necessary. Subsequently, the algorithm generated an overall risk of bias judgment based on certain criteria for the results in each domain. The assessment was primarily conducted by two reviewers (JS and RuS), and any controversial discrepancies were resolved by a third reviewer (JL). Kappa values were calculated for each of the five domains and overall judgment.

### 2.5 Data extraction

A preconditioned, standardized list was utilized for data abstraction from each included study, including RCT characteristics (author, country, and publication year), sample attributes (inclusion criteria, cancer type and stage, and sample size), interventions, controls, outcome measures, and follow-up time. Three reviewers participated in the process, with two responsible for extracting the data and the third facilitating a consensus view in case of discrepancies.

### 2.6 Data synthesis and analysis

Apart from the items mentioned above, for various outcome measures, we have implemented a standardized methodology for extracting or converting test statistics into mean ± standard deviation (M ± SD) across various outcome measures. Formulas involving SD = SE × $\sqrt{N}$ , SE = MD / t, t = f(x) = tinv (p, df), df = NE + NC - 2, SD = SE / $\sqrt[{2}]{\frac{1}{NE}\;+\;\frac{1}{NC}}$ (SE = standard error, N = sample size, MD = mean deviation, and df = degrees of freedom) were applied. The *t*-value was converted using Microsoft Excel with the TINV function. In addition, for outcomes reported as least square means (LSM) that could not be converted to mean values, we sourced the initial data from the supporting information of the articles or contacted the corresponding author via email.

Review Manager (RevMan) 5.4 was utilized to calculate the pooled standardized mean differences (SMDs) equal to Cohen’s *d*, allowing the comparison of effect sizes (ESs) throughout the outcomes to run this meta-analysis (The Cochrane Collaboration, 2020). According to the perspective of Cohen, the ES < 0.2, 0.2 ≤ ES < 0.5, 0.5 ≤ ES < 0.8, and ES ≥ 0.8 are regarded as negligible, small, medium, and large effects, respectively (Cohen, 1988). Heterogeneity test with the χ^2^ test and I^2^ statistic and subgroup analyses were also conducted using the software. The random-effects model representing the average intervention efficacy was applied to respond to a substantial statistical heterogeneity (I^2^ ≥ 50% and *p* ≤ 0.1) in analyses. Otherwise, we preferred to use a fixed-effects model that calculates the intervention effect estimate. For studies with more than one control group, such as ACT vs. behavioral activation (BA) vs. WL, outcome measures were divided into pairwise comparisons using different comparators.

Publication bias was assessed using a funnel plot that plotted the pooled SMD post-treatment against the standard error for the outcome measures. A symmetrical distribution of the scatters would indicate an absence of publication bias; otherwise a concern about the existing publication bias would be taken into consideration. Funnel plots are presented in Appendix C (Supplementary Figures S1, S2).

### 2.7 Assessment of evidence quality

The GRADE system was used to evaluate the certainty of each piece of evidence (Schünemann et al., 2013). The eight assessment criteria were divided into (1) downgrading factors (i.e., risk of bias, indirectness, inconsistency, imprecision, and publication bias) and (2) upgrading factors (i.e., large magnitude of effect, dose–response, and confounders that likely minimize the effect). One of the four grades (high, moderate, low, and very low) of evidence quality for each outcome was reached according to the study design and answers to each criterion. Considering that an RCT starts with a baseline rating of high quality, we preferred to apply operational criteria for downgrading the evidence, with no upgrading factors considered. These assessments reflected the degree of confidence in our effect estimates. GRADE evidence profiles were generated using GRADEpro GDT (2023). Two reviewers conducted independent assessments, and a third reviewer resolved discrepancies between their results.

## 3 Results

### 3.1 Search results

**Figure 1:**
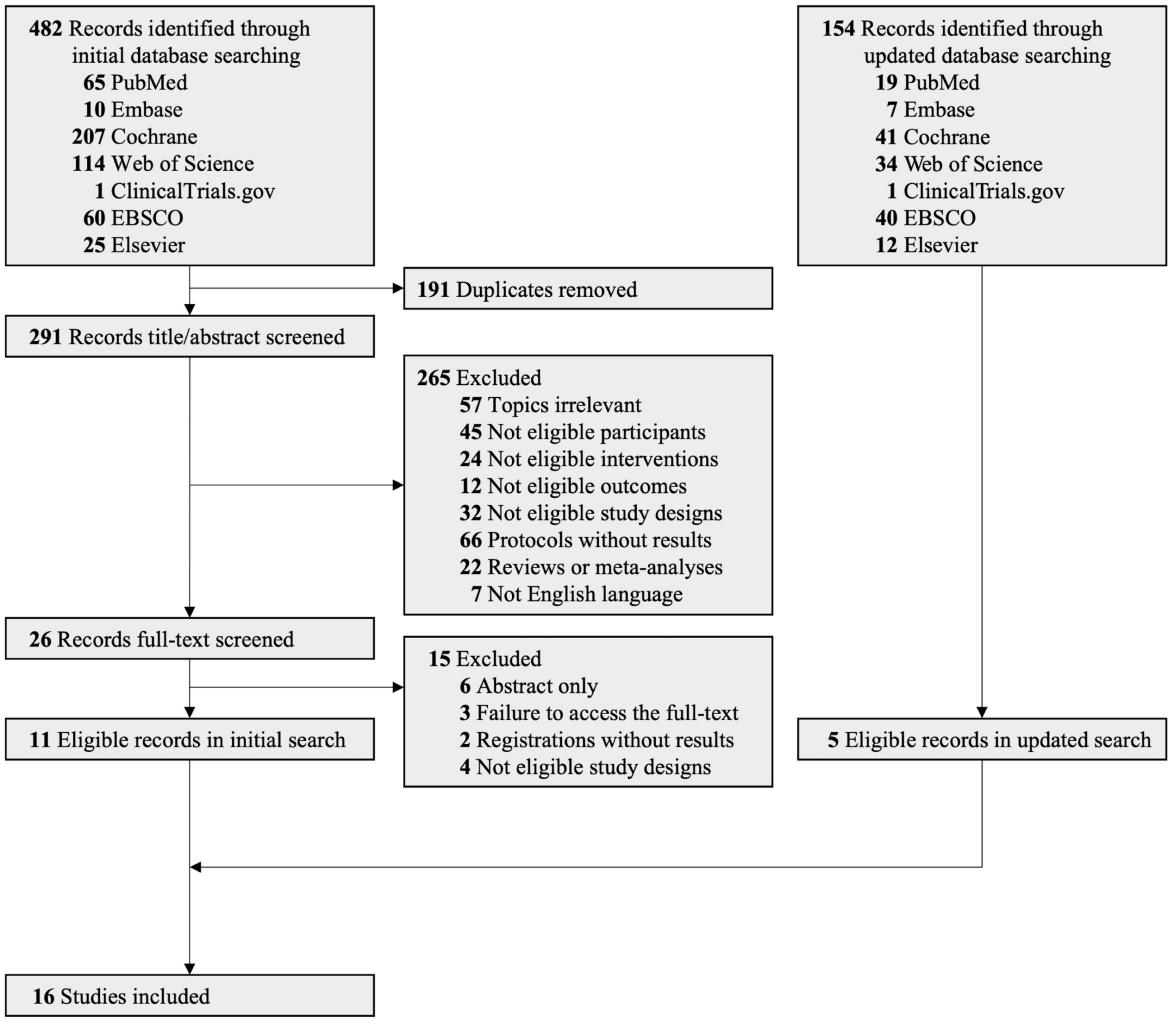
PRISMA flowchart of the selection procedure.

A PRISMA flowchart illustrating the literature retrieval and screening procedures is presented in Figure 1. Initially, 482 articles were identified from the database. After removing duplicates and studies with unqualified titles/abstracts, 26 publications were deemed potentially eligible for full-text retrieval. Among these, 11 studies without full content and 4 with ineligible study designs were excluded from the library. Therefore, 11 eligible RCTs were included in this meta-analysis (Rost et al., 2012; Mosher et al., 2018, 2019; Mani et al., 2019; Serfaty et al., 2019; Wells-Di Gregorio et al., 2019; Daneshvar et al., 2020; Johns et al., 2020; Fernández-Rodríguez et al., 2021; Ghorbani et al., 2021; Shari et al., 2021). The kappa coefficient between the two reviewers was 84.6% (*p* < 0.001) during full-text screening processes. The second supplementary search, conducted on 3 October 2023, yielded 5 additional eligible RCTs from 154 records across 7 databases (Li et al., 2022; Mosher et al., 2022; Peron, 2022; Burns et al., 2023; Wright et al., 2023). Finally, 16 eligible RCTs were included in this systematic review and meta-analysis (Rost et al., 2012; Mosher et al., 2018, 2019; Mani et al., 2019; Serfaty et al., 2019; Wells-Di Gregorio et al., 2019; Daneshvar et al., 2020; Johns et al., 2020; Fernández-Rodríguez et al., 2021; Ghorbani et al., 2021; Shari et al., 2021; Li et al., 2022; Mosher et al., 2022; Peron, 2022; Burns et al., 2023; Wright et al., 2023).

### 3.2 Risk of bias

**Figure 2:**
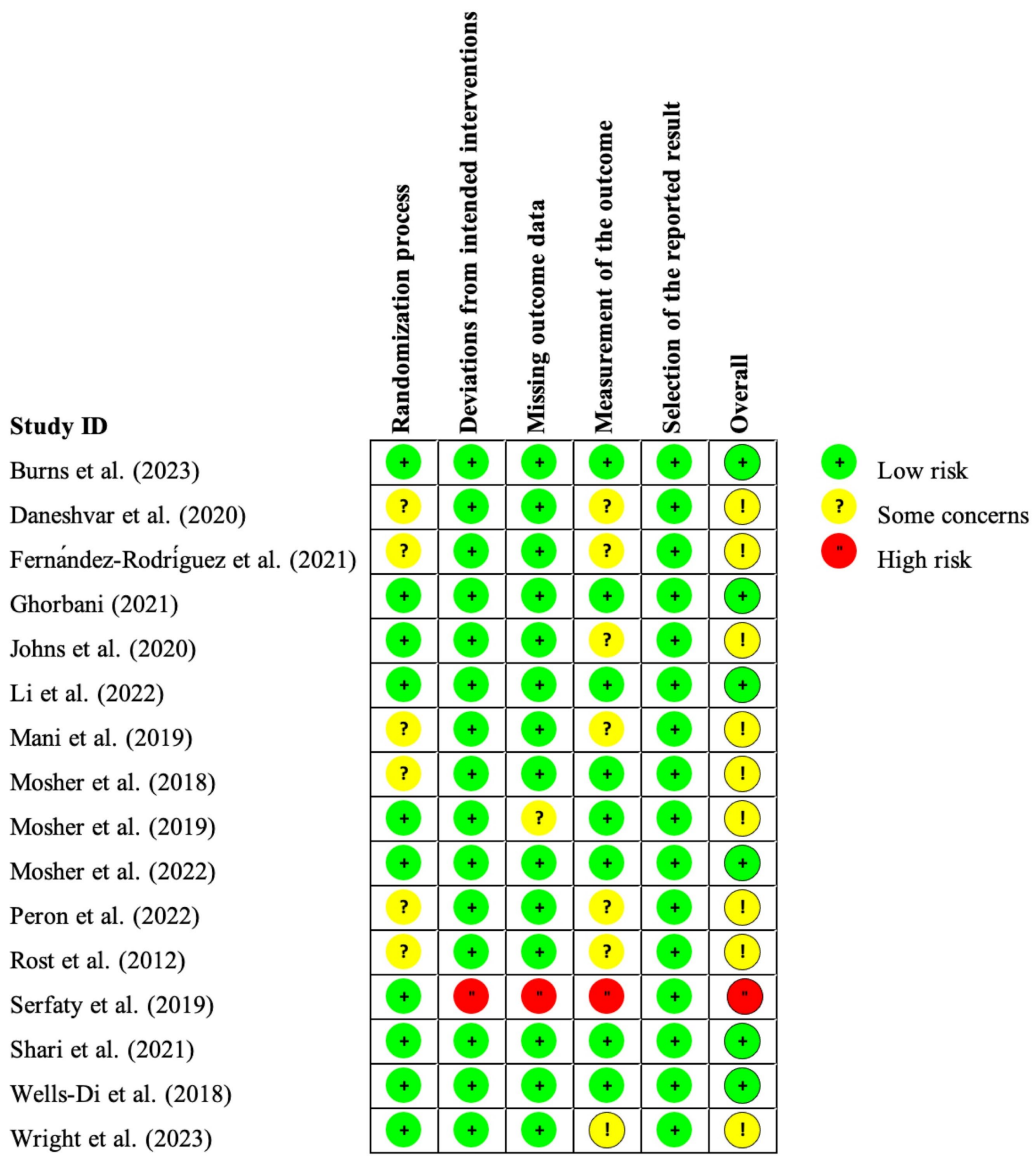
Summary of risk-of-bias judgments for each study.

**Figure 3:**
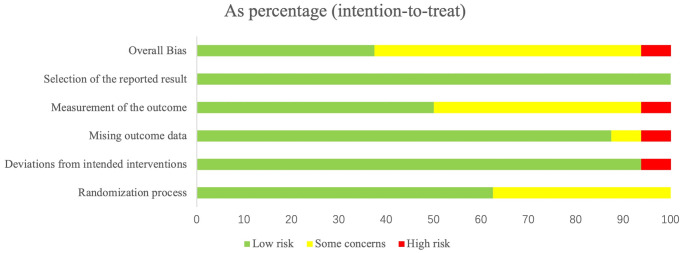
Summary of risk-of-bias judgments demonstrated as percentages across all included studies.

The Cochrane RoB assessment tool 2.0 was applied to evaluate the 16 eligible RCTs in this study (Higgins et al., 2016; Sterne et al., 2019). We chose the “intention-to-treat” option to investigate the efficacy of “assignment to intervention” during all evaluations. The results are summarized in Figures 2, 3.

As presented, six RCTs (38%) were deemed to be of high quality, with each of the six domains evaluated as low risk of bias; nine RCTs (56%) were rated into “some concerns” due to missing outcome data, imbalanced baseline measures (randomization process), or inappropriate measurement of the outcome; one RCT (6%) was judged to contain a high risk of bias mainly attributed to the insufficiently reported concealment of allocation sequence or the detailed information of it.

Notably, participant-reported outcomes were employed in the majority of the included RCTs, which makes it impossible to blind outcome assessors (also the study participant) for allocation in such psychotherapy studies. Under these circumstances, the results of the high risk of bias for these studies in this domain, the algorithm of mechanically generated tool, do not seem reasonable. Practically, participant-reported outcomes are extensively utilized in psychometry, and this method is perceived as acceptable (Higgins et al., 2019). Therefore, we rejudged involved studies in this domain as “some concerns” if appropriate participant-reported scales were applied for outcome measurements. Appendix B provides detailed information on the risk-of-bias assessment process for each study.

The kappa values in the six domains were calculated, ranging from 68.6% (*p* = 0.003) ~100% (*p* < 0.001), indicating an acceptable to nearly excellent consistency between the two assessors for risk of bias.

### 3.3 Study characteristics

Characteristics of the 16 RCTs are listed in Table 1. All studies were published within 5 years, except for Rost et al. (2012). Trials were conducted in the USA (*n* = 9) (Rost et al., 2012; Mosher et al., 2018, 2019; Wells-Di Gregorio et al., 2019; Johns et al., 2020; Mosher et al., 2022; Peron, 2022; Burns et al., 2023; Wright et al., 2023), Iran (*n* = 3) (Mani et al., 2019; Daneshvar et al., 2020; Ghorbani et al., 2021), China (*n* = 1) (Li et al., 2022), Spain (*n* = 1) (Fernández-Rodríguez et al., 2021), the UK (*n* = 1) (Serfaty et al., 2019), and Malaysia (*n* = 1) (Shari et al., 2021). The studies recruited patients no less than 18 years old with various cancer types, including breast cancer (*n* = 6) (Mosher et al., 2018; Mani et al., 2019; Daneshvar et al., 2020; Johns et al., 2020; Ghorbani et al., 2021; Shari et al., 2021), lung (*n* = 2) (Mosher et al., 2019; Li et al., 2022), ovarian (*n* = 2) (Rost et al., 2012; Wright et al., 2023), gastrointestinal cancer (*n* = 2) (Mosher et al., 2022; Burns et al., 2023), neurofibromatosis (*n* = 1) (Peron, 2022), and any type of cancer (*n* = 3) (Serfaty et al., 2019; Wells-Di Gregorio et al., 2019; Fernández-Rodríguez et al., 2021). A total of 16 RCTs involving 711 eligible subjects (355 in the ACT experimental group and 356 in the control group) were analyzed. The control conditions were divided into three categories: TAU (*n* = 4) (Rost et al., 2012; Johns et al., 2020; Li et al., 2022; Wright et al., 2023), WL (*n* = 5) (Wells-Di Gregorio et al., 2019; Fernández-Rodríguez et al., 2021; Ghorbani et al., 2021; Shari et al., 2021; Peron, 2022), and AC (*n* = 7) (group meetings and standardized talking controls) (Mosher et al., 2018, 2019; Mani et al., 2019; Serfaty et al., 2019; Daneshvar et al., 2020; Mosher et al., 2022; Burns et al., 2023). Despite the mostly utilized two-group parallel study design, two three-arm RCTs were conducted to explore the efficacy of ACT more comprehensively, which introduced the BA and survivorship education (SE) groups (Johns et al., 2020; Fernández-Rodríguez et al., 2021). The treatment interventions included the 6 core processes of ACT, and the number of sessions varied from 3 to 12, with each session lasting from 50 min to 2 h. The entire course of treatment lasted from 1 to 3 months. Ten of the 16 RCTs provided follow-up data with durations ranging from 1 to 6 months after the intervention (Mosher et al., 2018, 2019, 2022; Serfaty et al., 2019; Daneshvar et al., 2020; Johns et al., 2020; Fernández-Rodríguez et al., 2021; Ghorbani et al., 2021; Burns et al., 2023; Wright et al., 2023). Adherence rates were also investigated, with face-to-face designed ACT intervention with adherence rates of 35% ~ 100% at post-treatment and follow-up time-points in 8 RCTs (Rost et al., 2012; Mani et al., 2019; Serfaty et al., 2019; Daneshvar et al., 2020; Johns et al., 2020; Fernández-Rodríguez et al., 2021; Ghorbani et al., 2021; Shari et al., 2021), and ACT with other delivery methods (i.e., telephone and combined with media)-based trials demonstrating 70% ~ 100% adherence rates of patients to treatment at post-treatment and follow-up time-points in 8 RCTs (Mosher et al., 2018, 2019, 2022; Wells-Di Gregorio et al., 2019; Li et al., 2022; Peron, 2022; Burns et al., 2023; Wright et al., 2023).

**Table 1:** Characteristics of included studies.

Author	Inclusion criteria	Cancer type and stage	Interventions and sample Sizes	Control and sample size	Sessions × durations periods	Outcome measures	Follow-up time
Burns et al. (2023), USA	(1) ≥ 21 years old; (2) diagnosed with unresectable stage III or IV GI cancer; (3) FSI mean score ≥ 2.5; (4) no significant cognitive impairment; (5) ECOG score ≤ 2; (6) not receiving hospice care.	Gastrointestinal cancer; Stage III or IV	ACT (*n =* 20) Telephone-based Posttreatment *n* = 14 (lost 6) 70% Follow-up *n =* 14 (lost 6) 70%	Education/support (n = 20) Posttreatment *n =* 18 (lost 2) 90% Follow-up *n =* 15 (lost 5) 75%	6 × 50 min, 6w	PROMIS-A PROMIS-CC PROMIS-D PROMIS-SD PROMIS-PIa PROMIS-PIb FSI	3 M
Daneshvar et al. (2020), Iran	(1) 30–50 years of age; (2) being under chemotherapy; (3) literacy.	Breast cancer; NI	ACT (*n =* 15) Face-to-face Posttreatment *n =* 15 (lost 0) 100% Follow-up *n =* 15 (lost 0) 100%	Active-group meeting. (*n =* 15) Posttreatment *n =* 15 (lost 0) 100% Follow-up *n =* 15 (lost 0) 100%	8 × 1.5 h, 8w	RSS CBI	1 M
Fernández-Rodríguez et al. (2021), Spain	(1) between 18 and 65 years of age; (2) finished oncological treatment with surgery, chemotherapy, and/or radiotherapy for any type of malignant tumor; (3) currently be free of any type of oncological disease; (4) scores ≥8 in at least one of the subscales of the HADS	Any type of malignant tumor; NI	ACT (*n =* 17) Face-to-face Posttreatment *n =* 12 (lost 5) 70.6% Follow-up *n =* 12 (lost 5) 70.6%	WL (*n =* 27) Posttreatment *n =* 23 (lost 4) 85.2% Follow-up *n =* 17 (lost 10) 63.0%	12 × 1.5 h, 12w	HADS-T HADS-A HADS-D EROS BDI-IA-SCA BADS-T AAQ-2 CSQ-8	3 M
Ghorbani et al. (2021), Iran	(1) having a diagnosis of breast cancer by a physician; (2) not presenting other serious diseases (chronic obstructive pulmonary disease, pulmonary disease, diabetes, etc.); (3) being at least 18 years old; (4) having at least primary school education level; (5) being married; (6) being motivated to participate in the program; (7) having a depression score ≥ 10 according to the Depression, Anxiety and Stress Scale (DASS-21); (8) having an anxiety score ≥ 8 according to DASS-21 test; and (9) having no history of hospitalization in psychiatric section.	Breast cancer; NI	ACT (*n =* 20) Face-to-face Posttreatment *n =* 20 (lost 0) 100% Follow-up *n =* 20 (lost 0) 100%	WL (*n =* 20) Posttreatment *n =* 20 (lost 0) 100% Follow-up *n =* 20 (lost 0) 100%	8 × 1.5 h, 8w	DASS-21 AAQ-2 CPAQ	2 M
Johns et al. (2020), USA	(1) aged ≥18 years, (2) had stage I to stage III breast cancer, (3) had completed curative treatment (ongoing endocrine therapy was allowed), (4) had not experienced a cancer recurrence, and (5) had clinically significant FCR (Fear of Cancer Recurrence Inventory–Short Form [FCRI-SF] 8 scores ≥13)	Breast cancer; Stage I to Stage III	ACT (*n =* 33) Face-to-face Posttreatment *n =* 29 (lost 4) 87.9% Follow-up *n =* 30 (lost 3) 90.9%	TAU (*n =* 26) Posttreatment *n =* 25 (lost 1) 96.2% Follow-up *n =* 25 (lost 1) 96.2%	6 × 2 h, 6w	FCRI-PDS GAD-7 PHQ-8 Cancer-AAQ FCRI-FIS PROMIS-P PROMIS-M IES-R	6 M
Li et al. (2022), China	(1) ≥ 18 years old; (2) diagnosed with stage III/IV lung cancer via pathological section or cytology; (3) FSI score ≥ 3; (4) had a reliable Internet connection and a mobile smartphone.	Lung cancer; Stage III/IV	ACT (*n =* 20) Face-to-face and Internet-based. Posttreatment *n =* 15 (lost 5) 75%	TAU (*n =* 20) Posttreatment *n =* 20 (lost 0) 100%	4 × 1 ~ 1.5 h, 4w	FSI FACT-L MFI PHQ-9 GAD-7 IES-R	\
Mani et al. (2019), Iran	(1) no major psychiatric disorders, (2) at least a primary school education, (3) having undergone treatment, (4) at least 3 months after disease diagnosis, and breast cancer stages 2 or 3.	Breast cancer; Stages II or III	ACT (*n =* 15) Face-to-face Posttreatment *n =* 15 (lost 0) 100% No follow-up	Active-group meeting (*n =* 15) Posttreatment *n =* 15 (lost 0) 100% No follow-up	8 × 2 h, 1 m	PANAS QOL AHS	\
Mosher et al. (2022), USA	(1) diagnosed with unresectable stage III or IV gastrointestinal cancer; (2) Fatigue Interference subscale of FSI mean score ≥ 2.5; and (3) a consenting family caregiver.	Gastrointestinal cancer; Stage III or IV	ACT (*n =* 20) Telephone-based Posttreatment *n* = 14 (lost 6) 70% Follow-up *n =* 14 (lost 6) 70%	Education/support (*n =* 20) Posttreatment *n =* 18 (lost 2) 90% Follow-up *n =* 15 (lost 5) 75%	6 × 50 min, 6w	FSI Zarit PROMIS VQ AAQ-2 MQoL	3 M
Mosher et al. (2019), USA	(1) a diagnosis of advanced lung cancer (i.e., stage III or IV non-small cell or extensive stage small cell lung cancer) at least 3 weeks before enrollment; (2) had at least one moderate-to-severe symptom, defined as a Rotterdam Symptom item score > 2 on a 1–4 scale for fatigue, pain, sleep disturbance, breathlessness, depressive symptoms, or worry; and (3) a consenting primary family caregiver	Advanced lung cancer; Stage III or IV	ACT (*n =* 25) Telephone-based Posttreatment *n =* 20 (lost 5) 80% Follow-up *n =* 20 (lost 5) 80%	Active-group meeting (*n =* 25) Posttreatment *n =* 18 (lost 7) 72% Follow-up *n =* 18 (lost 7) 72%	6 × 50 min, 6w	PROMIS-D PROMIS-A PROMIS-DT FSI PROMIS-F PROMIS-SRI PROMIS-PIa MDASI MSASB	6 W
Mosher et al. (2018), USA	(1) patients had been diagnosed with stage IV breast cancer at least 3 weeks prior to enrollment and (2) had at least one moderate-to-severe symptom, defined by T-scores ≥ (at least one-half standard deviation above the population mean) on a three-item Patient Reported Outcomes Measurement Information System (PROMIS) measure of pain severity or a four-item PROMIS measure of fatigue, sleep disturbance, depressive symptoms, or anxiety	Breast cancer; Stage IV	ACT (*n =* 23) Telephone-based Posttreatment *n =* 18 (lost 5) 78.3% Follow-up *n =* 17 (lost 6) 73.9%	Active-group meeting (*n =* 24) Posttreatment *n =* 21 (lost 3) 87.5% Follow-up *n =* 20 (lost 4) 83.3%	6 × 1 h, 6w	PROMIS-D PROMIS-A FSI PROMIS-F PROMIS-SRI PROMIS-SD PROMIS-PIa MDASI	6 W
Peron (2022), USA	(1) Aged 19 ~ 59; (2) Diagnosis of neurofibromatosis type 1 (NF1) through germline mutation OR clinical diagnosis; (3) Possession of a plexiform neurofibromas (PN) documentation, based on either clinical exam or imaging; (4) Mean score of the Pain Interference Index ≥2.0 with self-report of chronic pain interfering daily functions at least 3 months. (5) Regular access to the Internet; (6) Ability to understand and the willingness to sign a written informed consent document. (7) No anticipated major changes in their pain treatment regimen or enrollment in a new treatment study presumed to impact pain soon. (8) Comprehension of the English language.	Neurofibromatosis type 1/ Plexiform Neurofibromas; NI	ACT (*n =* 32) Face-to-face plus Internet-based Posttreatment *n =* 32 (lost 0) 100% Follow-up *n =* 30 (lost 2) 93.8%	WL (*n =* 34) Posttreatment *n =* 30 (lost 4) 88.2% Follow-up *n =* 30 (lost 4) 88.2%	5 × 2 h, 8w	PROMIS-PIa PedsQL CPAQ NRS-11 CES-D PASS-20 EKG PIPS	\
Rost et al. (2012), USA	Those labeled as having Stage III or IV ovarian cancer were approached by an experimenter when they checked into the clinic or were waiting to see their oncologist while in the waiting room or exam room.	Ovarian cancer; Stage III or IV	ACT (*n =* 25) Face-to-face Posttreatment *n =* 15 (lost 10) 60% No follow-up	TAU (*n =* 22) Posttreatment *n =* 16 (lost 6) 72.7% No follow-up	12 × 1 h, 12w	BDI-II BAI POMS CECS WBSI COPE FACT-G	\
Serfaty et al. (2019), UK	People with advanced cancer attending day-therapy services, as in or out-patients, at three hospices in London, UK, were considered for participation if they were aged 18 years or more with a diagnosis of advanced cancer not amenable to cure (i.e., metastases at first diagnosis, subsequent recurrence, or lung cancer with or without metastases) FACT-G score below 81.	Any type of advanced cancers; Stage III or IV	ACT (*n =* 20) Face-to-face Posttreatment *n =* 7 (lost 13) 35% Follow-up *n =* 7 (lost 13) 35%	Active-group meeting (*n =* 22) Posttreatment *n =* 11 (lost 11) 50% Follow-up *n =* 8 (lost 14) 36.4%	8 × 1 h, 3 m	K10 AAQ-2 FACT-G 2-MWT 1-MSTST EQ-5D-5L	3 M
Shari et al. (2021), Malaysia	Breast cancer patients aged ≥18 years, had a reduction score from 6.9- to 10.6 of FACT-cog score from baseline and received standard adjuvant chemotherapy (FEC, FAC, taxane-based chemotherapy, and cyclophosphamide, methotrexate, fluorouracil (CMF))	Breast cancer; Stages I to III	ACT (*n =* 32) Face-to-face Posttreatment *n =* 30 (lost 2) 93.8% No follow-up	WL (*n =* 30) Posttreatment *n =* 30 (lost 0) 100% No follow-up	4 × 1 h, 12w	HADS-A HADS-D AAQ-2 FACT-F FACT-Cog	\
Wells-Di Gregorio et al. (2019), USA	Diagnosis of advanced cancer, sleep difficulties, and at least 18 years of age. Preliminary data from 141 advanced cancer patients suggested high levels of worry, depression, and fatigue, so we did not use these symptoms as inclusion criteria. We defined advanced cancer as a disease type with <55% chance of 5-year survival per SEER statistics in 2008–2009.	Any type of advanced cancer; Stages II to IV	CBT-ACT (17) Combined mode Posttreatment *n =* 14 (lost 3) 82.4% No follow-up	WL (*n =* 13) Posttreatment *n =* 11 (lost 2) 84.6% No follow-up	3 × 1.5 h, 6w	PSWQ CES-D STAI JSCS-Emot JSCS-Tot FSI NSFSD-SE NSFSD-SL ISI ESS	\
Wright et al. (2023), USA	(1) ≥ 18 years old; (2) diagnosed with advanced ovarian cancer; (3) received PARPi for ≥2 months; (4) English speaking; (5) ≥ 4 average rating on the first three items of the FSI 0–10 scale.	Ovarian cancer; Stages III/IV	REVITALIZE based on ACT Videoconference (21) Posttreatment n = 15 (lost 6) 71.4% Follow-up *n =* 15 (lost 6) 71.4%	TAU (*n =* 23) Posttreatment n = 23 (lost 0) 100% Follow-up n = 23 (lost 0) 100%	6 × 1 h, 6-8w	FSI GAD-7 PHQ-8 FCRI-SF FACT-O	1 M

AAQ-2, Acceptance and Action Questionnaire-II; AHS, Adult Hope Scale; BAI, The Beck Anxiety Inventory; BDI-II, Short form of Beck Depression Inventory-II; CBI, Cancer Behavior Inventory; CECS, The Courtland Emotional Control Scale; CES-D, Center for Epidemiological Studies Depression Scale; CPAQ, Chronic Pain Acceptance Questionnaire; DASS-21, Depression, Anxiety, and Stress Scale; EROS, Environmental Reward Observation Scale; EKG, Electrocardiogram; ESS, Epworth Sleepiness Scale (Daytime Sleepiness); FACT, Functional Assessment of Cancer Therapy; FACT-G, FACT-General, FACT-Cog, FACT-Cognitive, FACT-L, Functional Assessment of Cancer Therapy–Lung, Version 4, FCRI-Functioning Impairments Subscale, FACT-O, Functional Assessment of Cancer Therapy-Ovarian Cancer; FCRI-PDS, FCRI-Psychological Distress Subscale; FCRI-FIS, FCRI-Functioning Impairments Subscale; FSI, Fatigue Symptom Inventory; GAD-7, Generalized Anxiety Disorder Scale; HADS, Hospital Anxiety and Depression Scale; HADS-A, Anxiety Subscale of HADS; HADS-D, Depression Subscale of HADS; IES-R, Impact of Events Scale-Revised; ISI, the Insomnia Severity Index; JSCS-Emot, James Supportive Care Screening—Emotional Distress; JSCS-Tot, James Supportive Care Screening—Cancer-Related Distress; K10, The Kessler Psychological Distress Scale; MDASI, MD Anderson Symptom Inventory; MFI, The Multidimensional Fatigue Inventory; MQoL, McGill Quality of Life Questionnaire-Revised; MSASB, Memorial Symptom Assessment Scale (Breathlessness); NRS-11, Numeric Rating Scale-11; NSFSD-SE, National sleep Foundation Sleep Diary (Sleep Efficiency); NSFSD-SL, National Sleep Foundation Sleep Diary (Sleep Latency); PANAS, Positive and Negative Affect Schedule; PASS-20, Pain Anxiety Assessment Scale-20; PANAS, Positive and Negative Affect Schedule; PedsQL, Pediatric Quality of Life Inventory; PHQ-9, The Patient Health Questionnaire; PIPS, 12-item Psychological Inflexibility in Pain Scale; POMS, the Profile of Mood States (Distress); PROMIS, Patient-Reported Outcomes Measurement Information System; PROMIS-A, PROMIS anxiety; PROMIS-CC, PROMIS cognitive concerns; PROMIS-D, PROMIS depression, PROMIS-DT, PROMIS distress thermometer, PROMIS-F, PROMIS fatigue, PROMIS-P, PROMIS Global Health Scale-Physical; PROMIS-M, PROMIS Global Health Scale-Mental, PROMIS-PIa, PROMIS scale for pain interference, PROMIS-PIb, PROMIS scale for pain intense; PROMIS-SRI, PROMIS scale for sleep-related impairment, PROMIS SD, four-item PROMIS short-form sleep disturbance, PSWQ, Penn State Worry Questionnaire; QoL, Quality of Life Questionnaire; RSS, Rosenberg Self-esteem Scale; STAI, the State–Trait Anxiety Inventory; VQ, Valuing Questionnaire; WBSI, The White Bear Thought Suppression Inventory; 2-MWT, Two-Minute Walking Test; 1-MSTST, One-Minute Sit-to-Stand Test; NI, No information.

### 3.4 Effect of ACT on patients with cancer

Based on the postintervention and follow-up time-points, we performed a meta-analysis to investigate the effect of ACT on patients with cancer. The corresponding pooled ESs, 95% confidence intervals (*CI*), numbers of included studies and participants, I^2^ values, and weights are illustrated in the figures. Table 2 summarizes the results of the meta-analysis and the GRADE.

**Table 2:** Meta-analytic results and GRADE of ACT on cancer patients.

Analyses	Time-points	Effect estimate (95% CI)	No. of studies	No. of participants		I^2^ (%)	Quality of evidence (domains of downgrading)
				Intervention	Control		
Anxiety	Postintervention	−0.41 (−0.71, −0,11)	11	258	256	65	⊕ ⊕ ⊕ Moderate (c↓)
	Follow-up	−0.37 (−0.66, −0.08)	6	134	135	29	⊕ ⊕ ⊕ Moderate (c↓)
Depression	Postintervention	−0.45 (−0.63, −0.27)	11	258	256	49	⊕ ⊕ ⊕ Moderate (c↓)
	Follow-up	−0.52 (−0.77, −0.28)	6	134	135	0	⊕ ⊕ ⊕ Moderate (c↓)
QoL	Postintervention	0.24 (0.02, 0.47)	7	158	152	20	⊕ ⊕ ⊕ Moderate (c↓)
	Follow-up	0.14 (−0.18, 0.46)	4	81	77	49	⊕ ⊕ ⊕ Moderate (c↓)
Fatigue	Postintervention	−0.04 (−0.30, 0.23)	8	176	175	38	⊕ ⊕ ⊕ Moderate (c↓)
	Follow-up	−0.11 (−0.51, 0.29)	5	109	112	55	⊕ ⊕ ⊕ Moderate (c↓)
Insomnia	Postintervention	0.17 (−0.18, 0.52)	5	105	102	39	⊕ ⊕ ⊕ Moderate (c↓)
	Follow-up	0.20 (−0.09, 0.50)	4	88	89	0	⊕ ⊕ ⊕ Moderate (c↓)
Pain	Postintervention	−0.20 (−0.72, 0.33)	5	120	119	75	⊕ ⊕ Low (b↓, c↓)
	Follow-up	−0.09 (−0.70, 0.51)	4	88	89	75	⊕ ⊕ Low (b↓, c↓)
Global symptom	Postintervention	−0.07 (−0.38, 0.25)	3	81	75	0	⊕ ⊕ ⊕ Moderate (c↓)
	Follow-up	−0.14 (−0.45, 0.18)	3	81	75	0	⊕ ⊕ ⊕ Moderate (c↓)
AAQ-2	Postintervention	−0.81 (−1.50, −0.11)	5	115	119	84	⊕ ⊕ Low (b↓, c↓)
	Follow-up	−0.71 (−1.12, −0.31)	4	85	83	38	⊕ ⊕ ⊕ Moderate (c↓)

95% CI: 95% confidence interval, NA: not applicable. Reasons for specific upgrading and downgrading: a. Risk of bias. b. Inconsistence. c. Indirectness. d. Imprecision. e. Publication bias. f. Large effect. g. Plausible confounding. f. Dose–response gradient.

#### 3.4.1 Anxiety

**Figure 4:**
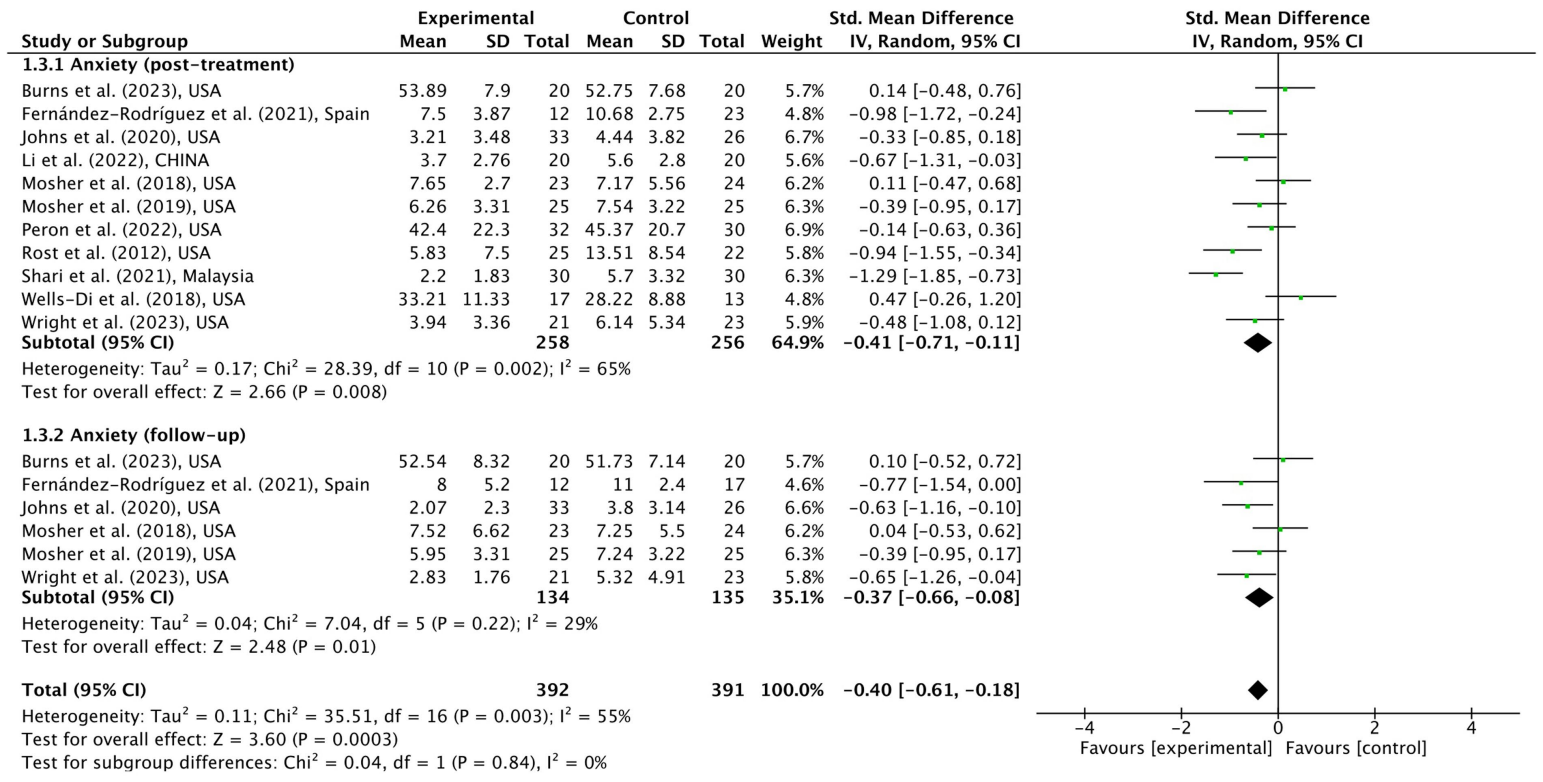
Meta-analytic results of ACT for cancer patients on anxiety in post-intervention and follow-up time-points.

Eleven studies provided data on anxiety, of which six reported postintervention and follow-up results (Rost et al., 2012; Mosher et al., 2018, 2019; Wells-Di Gregorio et al., 2019; Johns et al., 2020; Fernández-Rodríguez et al., 2021; Shari et al., 2021; Li et al., 2022; Peron, 2022; Burns et al., 2023; Wright et al., 2023). Figure 4 shows that significant reductions in anxiety in ACT treatment participants were observed at the post-treatment time-point [SMD = −0.41 (95% CI, −0.71, −0.11); *p* = 0.008; I^2^ = 65%] and follow-up time-point [SMD = −0.37 (95% CI, −0.66, −0.08); *p* = 0.01; I^2^ = 29%] when compared with control groups. Both ESs were considered small.

#### 3.4.2 Depression

**Figure 5:**
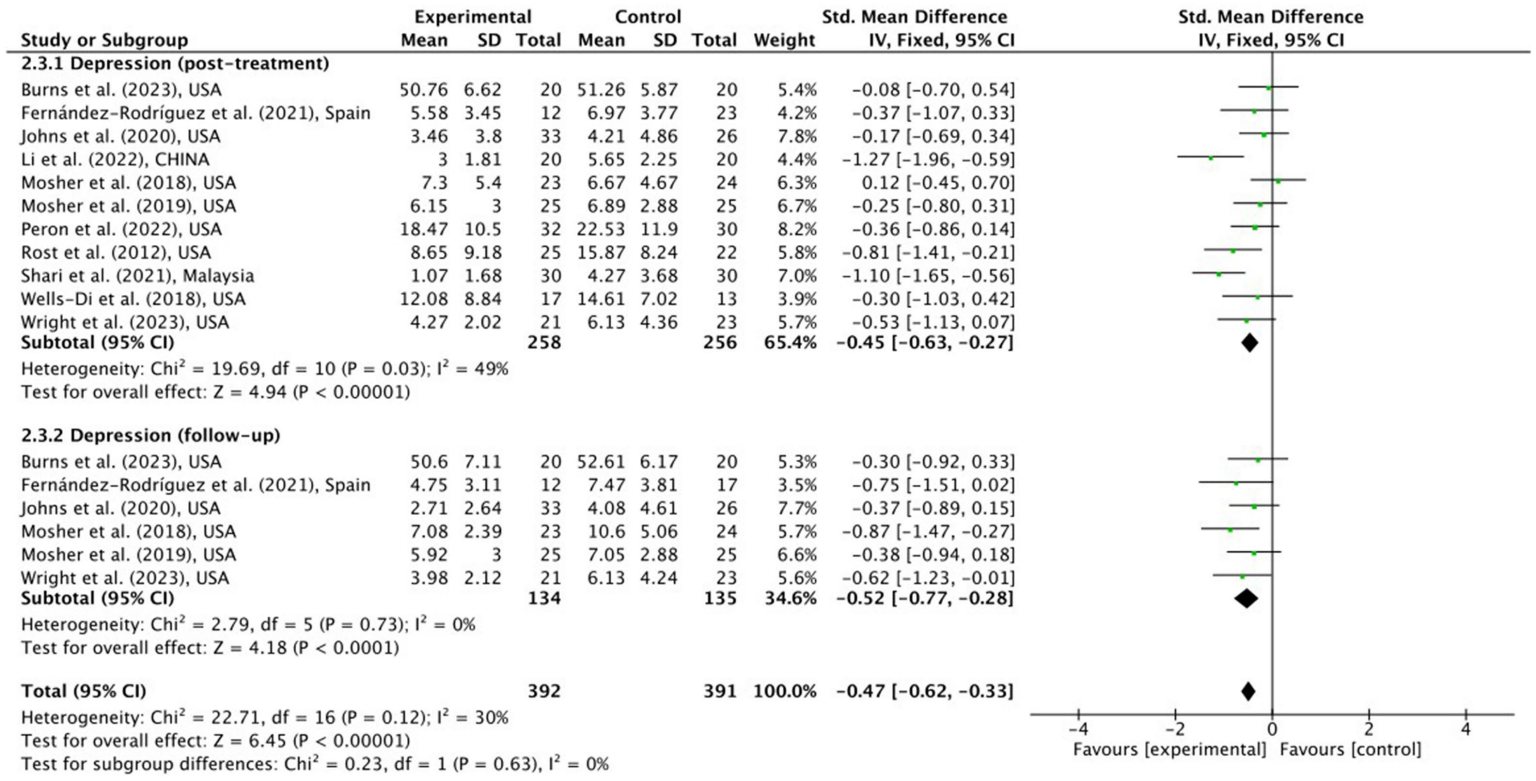
Meta-analytic post-treatment result of ACT for cancer patients on depression in post-intervention and follow-up time-points.

Similarly, 11 RCTs reported depression data, and 6 conducted a follow-up investigation (Rost et al., 2012; Mosher et al., 2018, 2019; Wells-Di Gregorio et al., 2019; Johns et al., 2020; Fernández-Rodríguez et al., 2021; Shari et al., 2021; Li et al., 2022; Peron, 2022; Burns et al., 2023; Wright et al., 2023). From Figure 5, patients treated with ACT demonstrated significantly lower depression levels in post-treatment [SMD = −0.45 (95% CI, −0.63, −0.27); *p* < 0.001; I^2^ = 49%] and follow-up [SMD = −0.52 (95% CI, −0.77, −0.28); *p* < 0.001; I^2^ = 0%] without heterogeneity.

#### 3.4.3 QoL

Nine studies evaluated QoL, five of which had follow-up data (Rost et al., 2012; Mani et al., 2019; Serfaty et al., 2019; Daneshvar et al., 2020; Johns et al., 2020; Li et al., 2022; Mosher et al., 2022; Peron, 2022; Wright et al., 2023). The pooled ES values (Supplementary Figure S3) indicated significant difference between intervention and control groups at the post-treatment [SMD = 0.24 (95% CI, 0.02, 0.47); *p* = 0.03; I^2^ = 20%] but no difference at follow-up [SMD = 0.14 (95% CI, −0.18, 0.46); *p* = 0.38; I^2^ = 49%] time-points. Due to the high heterogeneity investigated by the leave-one-out analysis, data from Mani et al. (2019) and Daneshvar et al. (2020) were deleted.

#### 3.4.4 Other symptoms

Eleven studies evaluated at least one other symptom (Mosher et al., 2018, 2019, 2022; Wells-Di Gregorio et al., 2019; Johns et al., 2020; Ghorbani et al., 2021; Shari et al., 2021; Li et al., 2022; Peron, 2022; Burns et al., 2023; Wright et al., 2023), including fatigue (Mosher et al., 2018, 2019; Wells-Di Gregorio et al., 2019; Shari et al., 2021; Li et al., 2022; Mosher et al., 2022; Burns et al., 2023; Wright et al., 2023), insomnia (Mosher et al., 2018, 2019; Wells-Di Gregorio et al., 2019; Mosher et al., 2022; Burns et al., 2023), pain (Mosher et al., 2018, 2019; Ghorbani et al., 2021; Peron, 2022; Burns et al., 2023), and global symptoms (Mosher et al., 2018, 2019; Johns et al., 2020), with eight, five, five, and three studies reporting their efficacy in post-treatment, respectively. Supplementary Figure S4.1 shows the results for post-treatment, whereas Fig. S4.2 presents the follow-up findings with at least three studies in each subgroup. As forest plots illustrate, ACT may not be effective in reducing symptoms of fatigue [postintervention SMD = −0.04 (95% CI, −0.30, 0.23); *p* = 0.80; I^2^ = 38%; follow-up SMD = −0.11 (95% CI, −0.51, 0.29); *p* = 0.58; I^2^ = 55%], insomnia [postintervention SMD = 0.17 (95% CI, −0.18, 0.52); *p* = 0.35; I^2^ = 39%; follow-up SMD = 0.20 (95% CI, −0.09, 0.50); *p* = 0.18; I^2^ = 0%], and pain [postintervention SMD = −0.20 (95% CI, −0.72, 0.33); *p* = 0.46; I^2^ = 75%; follow-up SMD = −0.09 (95% CI, −0.70, 0.51); *p* = 0.77; I^2^ = 75%] in patients with cancer. All pooled meta-analyses’ ESs were non-significant.

#### 3.4.5 Psychological flexibility

Psychological flexibility was measured using the Acceptance and Commitment Questionnaires I/II. Six studies reported this outcome (Serfaty et al., 2019; Johns et al., 2020; Fernández-Rodríguez et al., 2021; Ghorbani et al., 2021; Shari et al., 2021; Mosher et al., 2022), of which five provided follow-up data (Serfaty et al., 2019; Johns et al., 2020; Fernández-Rodríguez et al., 2021; Ghorbani et al., 2021; Mosher et al., 2022). Supplementary Figure S5 illustrates that compared to controlled conditions, ACT significantly improved psychological flexibility in both postintervention [SMD = −0.81 (95% CI, −1.50, −0.11); *p* = 0.02; I^2^ = 84%] and follow-up [SMD = −0.71 (95% CI, −1.12, −0.31); *p* = 0.0006; I^2^ = 38%] time-points.

#### 3.4.6 Subgroup analyses

To further investigate the sources of heterogeneity and reduce the influence of confounding factors on the results, subgroup analyses of the efficacy on anxiety, depression post-treatment, and both time-points of the AAQ-2 results were performed according to the different control conditions (i.e., TAU, WL, and AC).

Four, four, and three RCTs reporting anxiety applied TAU (Rost et al., 2012; Johns et al., 2020; Li et al., 2022; Wright et al., 2023), WL (Fernández-Rodríguez et al., 2021; Shari et al., 2021; Peron, 2022), and AC (Mosher et al., 2018, 2019; Burns et al., 2023) as control strategies, respectively. Supplementary Figure S6 illustrates a medium, homogenous, and statistically significant ES [SMD = −0.58 (95% CI, −0.87, −0.29); *p* = 0.0001; I^2^ = 0%] favoring ACT over TAU. No significant differences were observed between WL and AC. The heterogeneity was detected in the WL control condition subgroup (I^2^ = 83%).

For depression (Supplementary Figure S7), moderate ESs were found in TAU [SMD = −0.66 (95% CI, −1.11, −0.21); *p* = 0.004; I^2^ = 56%] and WL [SMD = −0.56 (95% CI, −0.96, −0.16); *p* = 0.006; I^2^ = 42%] control condition subgroups with medium heterogeneity (Rost et al., 2012; Mosher et al., 2018, 2019; Wells-Di Gregorio et al., 2019; Johns et al., 2020; Fernández-Rodríguez et al., 2021; Shari et al., 2021; Li et al., 2022; Peron, 2022; Burns et al., 2023; Wright et al., 2023).

When compared with TAU [postintervention SMD = −0.56 (95% CI, −1.08, −0.03); *p* = 0.04; follow-up SMD = −0.75 (95% CI, −1.28, −0.22); *p* = 0.006] and WL [postintervention SMD = −1.12 (95% CI, −2.19, −0.06); *p* = 0.04; I^2^ = 87%; follow-up SMD = −0.88 (95% CI, −1.76, −0.00); *p* = 0.05; I^2^ = 66%] (Serfaty et al., 2019; Johns et al., 2020; Fernández-Rodríguez et al., 2021; Ghorbani et al., 2021; Shari et al., 2021; Mosher et al., 2022), ACT showed more efficiency in improving the psychological flexibility in patients with cancer (Supplementary Figure S8).

## 4 Discussion

This systematic review and meta-analysis aimed to evaluate the efficacy of ACT in treating psychological distress in patients with cancer. Compared to recent studies (Li H. et al., 2021; Li Z. H. et al., 2021; Zhao et al., 2021; Fang et al., 2023; Maunick et al., 2023; Zhang et al., 2023), this report has acceptable heterogeneity in the primary outcomes and focused on retrieving evidence from English-language randomized controlled trials to comprehensively investigate the potential effectiveness of ACT in different types of cancer. Furthermore, the GRADE approach had been applied to rate the quality of the evidence summarized (Schünemann et al., 2013). The results indicated statistically significant ESs in alleviating anxiety and depression and promoting QoL and cancer acceptance (measured by the AAQ-2). No significant ES was observed in the other patient symptoms.

Low to moderate quality of evidence suggests non-significant results in improving other symptoms such as fatigue, insomnia, pain, and global symptoms in patients with cancer. These results were partially consistent with those of previous studies (Fang et al., 2023; Zhang et al., 2023). Directly alleviating or eliminating physical symptoms like these is not the primary purpose of ACT. Instead, ACT reshapes the relationship with negative thoughts and feelings by fostering psychological flexibility, enabling patients to be free from psychological distress and pursue value-based living under the circumstances of accepting and coexisting with such symptoms. Considering pain intensity as an example attempts to reduce or eliminate the intrinsic chronic symptoms of the disease have been reported to be futile or even detrimental (Janssen et al., 2004; McCracken et al., 2007; Jensen et al., 2012; Bushnell et al., 2013; Hughes et al., 2017). Therefore, measurements of symptom acceptance have recently been preferred by researchers over intensity scales (Kerns et al., 2011; McCracken and Morley, 2014; McCracken and Vowles, 2014; Du et al., 2021). However, these findings are inconsistent with several evidence-based studies illustrating the efficacy of ACT in reducing pain intensity. These pooled ESs were often small and could be interpreted as additional effects of treatment.

Naturally, due to increased psychological flexibility, patients’ acceptance and related psychological distress, including anxiety and depression, significantly improved consistent with most ACT studies (Li H. et al., 2021; Li Z. H. et al., 2021; Zhao et al., 2021; Fang et al., 2023). Characterized by “openness, awareness, and active engagement to living,” psychology flexibility creates a functional contextual framework for patients to concentrate on being present and chasing value-based action to diminish the intensity, frequency, and, most importantly, the influence of psychological experiences (Bluett et al., 2014; Gloster et al., 2020). Additionally, the negative influence of psychological inflexibility, such as avoidance and cognitive fusion, was verified, further supporting the adaptive aspect of psychological flexibility (Wicksell et al., 2008; Levin et al., 2014). In agreement with several studies, ACT was qualified to facilitate patients’ acceptance of the status quo and reduce experiential avoidance according to the pooled results of the AAQ-2 in this systematic review and meta-analysis, despite the examined heterogeneity (Graham et al., 2016).

Various sources of heterogeneity, such as different control conditions, measures, cancer types, and delivery methods of ACT, may influence the reliability of estimates of pooled ESs despite the restrictions set in the PRISMA screening procedures (Andersson et al., 2014). Considering this, subgroup analyses of ACT efficacy on anxiety, depression, and psychological flexibility were performed to clarify the influence of various control strategies on the pooled ESs. The results revealed that ACT significantly reduced psychological distress levels and improved psychological flexibility in postintervention patients with cancer compared with TAU- and TAU/WL-treated patients in most cases. However, under certain circumstances, ACT may not be superior to AC methods, such as standardized meetings or other cognitive-behavioral therapies (Powers et al., 2009; Ost, 2014; A-Tjak et al., 2015; Hacker et al., 2016).

There are some similarities and differences in our findings compared to prior relevant systematic reviews and meta-analyses (Li H. et al., 2021; Li Z. H. et al., 2021; Zhao et al., 2021; Fang et al., 2023; Maunick et al., 2023; Zhang et al., 2023). On one hand, almost all studies support the association between ACT and the improvement of psychological flexibility and distress (anxiety and depression) in patients with cancer, which is due to the nature and purpose of ACT (Li H. et al., 2021; Li Z. H. et al., 2021; Zhao et al., 2021; Fang et al., 2023; Zhang et al., 2023). Four studies reported a significant result on QoL, which is consistent with this meta-analysis. On the other hand, some different results deserve attention. First, the non-significant result of fatigue is consistent with Zhang et al. (Zhang et al., 2023) but in contradiction to Fang et al. (2023) and Maunick et al. (2023), and one reason for the divergence is the different criteria of the included participants. The target population of Fang et al. (2023) and Maunick et al. (2023) is people with advanced cancer and with cancer plus chronic health conditions, which narrows and expands the scope of included participants, respectively compared to us. Second, in addition to these specific reasons, there is a common reason that also contributed to these divergences. In data extraction and synthesis processes, the mean and SD values of postintervention and follow-up time-points were utilized in this study, while others applied mean difference and mean SD between the two time-points results and baseline value. Through these similarities and differences, we hope to provide a new perspective in this field.

The quality of evidence was assessed using the GRADEpro GDT, which ranged from low to moderate (Schünemann et al., 2013). Of the meta-analytic results, 13 were moderate, and 3 were low. All the outcomes were downgraded for indirectness. Research shows that different ACT delivery methods (i.e., face-to-face, Internet, and telephone-based ACT) could have different retention rates and efficacies. Considering this, we regarded Internet- and telephone-based ACT as less “direct.” Three meta-analytic results involving pain and AAQ were downgraded for inconsistency due to I^2^ > 75% or non-overlapping 95% CI.

## 5 Limitations

This study has several limitations. On one hand, although subgroup analyses for different control strategies have been conducted, limited by article length and data size, potential sources of clinical or methodological heterogeneity exist in participants, intervention methods, measures, or RCT design, which the statistical I^2^ only partially reflects. For instance, participants with different cancer stages or treatments may experience different psychological distress and demands, leading to potential heterogeneity. Furthermore, as a cognitive-behavioral therapy with improved psychological flexibility, ACT itself is inherently flexible with different specific language materials for different trials despite the same course title or outline being shared, and the therapeutic efficacy of distinct delivery methods such as face-to-face, Internet-based, or even telephone-based ACT remains unclear (Andersson et al., 2014; Lappalainen et al., 2014; Carlbring et al., 2018). On the other hand, several ES estimates comprised a small number of primary studies, possibly resulting in an underpowered analysis. Therefore, the degree to which the outcomes presented in this systematic review and meta-analysis reflect an ideal endpoint remains controversial.

## 6 Implications

Considering these limitations, more comprehensive subgroup analyses are needed, especially those based on different population characteristics and different delivery methods of ACT. Future RCTs should follow the CONSORT and TIDieR checklists more rigorously to optimize the reporting quality with precise descriptions of the sample and intervention characteristics. Finally, an optimal ACT design specialized for patients with cancer requires continuous exploration and development.

Much of the evidence, including this study, suggests that ACT has a promising future, as it is well adapted to clinical settings, improves psychological flexibility, and reduces psychological distress, such as anxiety and depression. It is of great value for relevant clinicians and teams to utilize these evidence bases to benefit patients with cancer and further develop clinical psychological care.

## 7 Conclusion

In this systematic review and meta-analysis, ACT was found to be more associated with improvements in patients with cancer on anxiety compared to treatments as usual and on depression and psychological flexibility compared to both treatments as usual and wait-list controls. However, ACT may not be superior to active controls such as standardized education or other CBTs. These findings provide a theoretical basis for future clinical practice. Limitations, such as insufficient studies and potential heterogeneity, may influence the reliability of ES estimates. In summary, ACT shows promise for alleviating cancer-related psychological distress; however, more high-quality RCTs with larger sample sizes are needed.

## Data availability statement

The original contributions presented in the study are included in the article/Supplementary material, further inquiries can be directed to the corresponding authors.

## Author contributions

XJ and JS had full access to all the data in the study and took responsibility for the integrity of the data and the accuracy of the data analysis, are co-first authors, and were involved in the conception and design of the study, drafted the manuscript, supervised the study. XJ, JS, RuS, YW, JL, and RoS contributed to the acquisition, analysis, or interpretation of data. XJ, JS, RuS, YW, JL, and RoS critically revised the manuscript for important intellectual content. JS, XJ, JL, RuS, RoS, and YW were involved in statistical analysis. XJ, RuS, and RoS extended administrative, technical, or material support. All authors contributed to the article and approved the submitted version.

## Footnotes

^1^https://hiru.mcmaster.ca/hiru/HIRU_Hedges_home.aspx
