# Supplementary material for: Acceptance and commitment therapy reduces psychological distress in patients with cancer: a systematic review and meta-analysis of randomized controlled trials
Source: Front Psychol. 2024 Jan 5;14:1253266. doi: 10.3389/fpsyg.2023.1253266 (PMC10796538; doi:10.3389/fpsyg.2023.1253266)
Supplement: Supplementary file 1 [file Table_1.DOCX]

Supplementary Material

# Appendix A: The Primary Search Strategies and Results.

**PubMed**: Search: (((“Neoplasms”[MeSH]) OR ((((((((((((((((((Neoplasms[Title/Abstract]) OR (Tumor[Title/Abstract]) OR (Tumors[Title/Abstract]) OR (Neoplasm[Title/Abstract]) OR (Neoplasia[Title/Abstract]) OR (Neoplasias[Title/Abstract]) OR (Cancer[Title/Abstract]) OR (Cancers[Title/Abstract]) OR (Malignant Neoplasm[Title/Abstract]) OR (Malignancy[Title/Abstract]) OR (Malignancies[Title/Abstract]) OR (Malignant Neoplasms[Title/Abstract]) OR (Neoplasm, Malignant[Title/Abstract]) OR (Neoplasms, Malignant[Title/Abstract]) OR (Benign Neoplasm[Title/Abstract]) OR (Benign Neoplasms[Title/Abstract]) OR (Neoplasm, Benign[Title/Abstract]) OR (Neoplasms, Benign[Title/Abstract]))) AND ((“Acceptance and Commitment Therapy”[MeSH] OR (Acceptance Commitment) OR (Acceptance Commitment Therapy)))) AND (Randomized Controlled Trial[Publication Type]) OR Randomized[Title/Abstract] OR Placebo[Title/Abstract].

| **PubMed** | | | |
| --- | --- | --- | --- |
| **NO.** | **Query** | **Results** | **Time** |
| #8 | Search: **#3 and #6 and #7** | 65 | 13:05:22 |
| #7 | Search: **randomized controlled trial[Publication Type] OR randomized[Title/Abstract] OR placebo[Title/Abstract]** | 924,595 | 13:01:06 |
| #6 | Search: **#4 or #5** | 4,963 | 12:37:19 |
| #5 | Search: **(Acceptance commitment) OR (acceptance commitment therapy)** | 4,963 | 12:15:32 |
| #4 | Search: **"Acceptance and Commitment Therapy"[Mesh]** Sort by: **Most Recent** | 623 | 12:09:32 |
| #3 | Search: **#1 or #2** | 4,509,337 | 12:00:46 |
| #2 | Search: **(((((((((((((((((Neoplasms[Title/Abstract]) OR (Tumor[Title/Abstract])) OR (Tumors[Title/Abstract])) OR (Neoplasm[Title/Abstract])) OR (Neoplasia[Title/Abstract])) OR (Neoplasias[Title/Abstract])) OR (Cancer[Title/Abstract])) OR (Cancers[Title/Abstract])) OR (Malignant Neoplasm[Title/Abstract])) OR (Malignancy[Title/Abstract])) OR (Malignancies[Title/Abstract])) OR (Malignant Neoplasms[Title/Abstract])) OR (Neoplasm, Malignant[Title/Abstract])) OR (Neoplasms, Malignant[Title/Abstract])) OR (Benign Neoplasms[Title/Abstract])) OR (Benign Neoplasm[Title/Abstract])) OR (Neoplasms, Benign[Title/Abstract])) OR (Neoplasm, Benign[Title/Abstract])** | 3,164,612 | 11:58:22 |
| #1 | Search: **"Neoplasms"[Mesh]** Sort by: **Most Recent** | 3,581,001 | 09:37:56 |

| **Embase** | | | |
| --- | --- | --- | --- |
| **Session results** | | | |
| **NO.** | **Query results** | **Results** | **Date** |
| #25. | #19 AND #23 AND #24 | 10 | 10 Dec 2021 |
| #24. | 'random':ab,ti OR 'placebo':ab,ti OR 'double-blind':ab,ti | 761,207 | 10 Dec 2021 |
| #23. | #20 OR #21 OR #22 | 1,885 | 10 Dec 2021 |
| #22. | 'acceptance commitment therapy':ti,ab | 53 | 10 Dec 2021 |
| #21. | 'acceptance commitment':ti,ab | 69 | 10 Dec 2021 |
| #20. | 'acceptance and commitment therapy'/exp | 1,842 | 10 Dec 2021 |
| #19. | #1 OR #2 OR #3 OR #4 OR #5 OR #6 OR #7 OR #8 OR #9 OR #10 OR #11 OR #12 OR #13 OR #14 OR #15 OR #16 OR #17 OR #18 | 6,190,867 | 10 Dec 2021 |
| #18. | 'neoplasm, benign':ti,ab | 62 | 10 Dec 2021 |
| #17. | 'neoplasms, benign':ti,ab | 159 | 10 Dec 2021 |
| #16. | 'benign neoplasm':ti,ab | 2,505 | 10 Dec 2021 |
| #15. | 'benign neoplasms':ti,ab | 18,289 | 10 Dec 2021 |
| #14. | 'neoplasms, malignant':ti,ab | 83 | 10 Dec 2021 |
| #13. | 'neoplasm, malignant':ti,ab | 64 | 10 Dec 2021 |
| #12. | 'malignant neoplasms':ti,ab | 34,430 | 10 Dec 2021 |
| #11. | 'malignancies':ti,ab | 205,011 | 10 Dec 2021 |
| #10. | 'malignancy':ti,ab | 247,236 | 10 Dec 2021 |
| #9. | 'malignant neoplasm':ti,ab | 7,487 | 10 Dec 2021 |
| #8. | 'cancers':ti,ab | 425,163 | 10 Dec 2021 |
| #7. | 'cancer':ti,ab | 2,698,793 | 10 Dec 2021 |
| #6. | 'neoplasias':ti,ab | 8,158 | 10 Dec 2021 |
| #5. | 'neoplasia':ti,ab | 79,870 | 10 Dec 2021 |
| #4. | 'neoplasm':ti,ab | 88,488 | 10 Dec 2021 |
| #3. | 'tumors':ti,ab | 892,563 | 10 Dec 2021 |
| #2. | 'tumor':ti,ab | 1,744,970 | 10 Dec 2021 |
| #1. | 'neoplasm'/exp | 5,399,377 | 10 Dec 2021 |

| **The Cochrane Library** | | | |
| --- | --- | --- | --- |
| **Search Name** | | Acceptance and Commitment Therapy | |
| **Last Saved** | | 10/12/2021 19:37:24 | |
| **ID** | **Search** | | **Results** |
| #1 | MeSH descriptor: [Neoplasms] explode all trees | | 85354 |
| #2 | (Tumor):ti,ab,kw OR (Tumors):ti,ab,kw OR (Neoplasm):ti,ab,kw OR (Neoplasia):ti,ab,kw OR (Neoplasias):ti,ab,kw (Word variations have been searched) | | 138425 |
| #3 | (Cancer):ti,ab,kw OR (Cancers):ti,ab,kw OR (malignant neoplasm):ti,ab,kw OR (Malignancy):ti,ab,kw OR (Malignancies):ti,ab,kw (Word variations have been searched) | | 188887 |
| #4 | (Malignant Neoplasms):ti,ab,kw OR (Neoplasm, Malignant):ti,ab,kw OR (Neoplasms, Malignant):ti,ab,kw OR (Benign Neoplasms):ti,ab,kw OR (Benign Neoplasm):ti,ab,kw (Word variations have been searched) | | 13106 |
| #5 | (Neoplasms, Benign):ti,ab,kw OR (Neoplasm, Benign):ti,ab,kw (Word variations have been searched) | | 3174 |
| #6 | #1 or #2 or #3 or #4 or #5 | | 238103 |
| #7 | MeSH descriptor: [Acceptance and Commitment Therapy] explode all trees | | 241 |
| #8 | (acceptance commitment):ti,ab,kw OR (acceptance commitment therapy):ti,ab,kw (Word variations have been searched) | | 1806 |
| #9 | #7 or #8 | | 1806 |
| #10 | #6 and #9 | | 209 |

| **Web of Science** | | |
| --- | --- | --- |
| **NO.** | **Query** | **Results** |
| #1 | TS=(Neoplasm* or Tumor or Tumor* or Neoplasm or Neoplasia or Neoplasia* or Cancer or Cancer* or Malignant Neoplasm or Malignancy or Malignancie* or Malignant Neoplasm* or Neoplasm, Malignant or Neoplasm*, Malignant or Benign Neoplasm* or Benign Neoplasm or Neoplasm*, Benign or Neoplasm, Benign) | 6,701,543 |
| #2 | TS=(acceptance commitment) | 5,511 |
| #3 | TS=(random* controlled trial OR random* OR placebo) | 2,596,890 |
| #4 | #3 AND #2 AND #1 | 114 |

| **ClinicalTrials.gov** | | |
| --- | --- | --- |
| **Terms and Synonyms Searched** | | |
| **Terms** | Search Results* | Entire Databases** |
| - Synonyms |  |  |
| **Neoplasms** | 1 study | 86,171 studies |
| - Neoplastic Syndrome | 1 study | 686 studies |
| - Cancer | -- | 51,546 studies |
| - Malignancy | -- | 3,572 studies |
| - Neoplasia | -- | 706 studies |
| - Neoplastic Disease | -- | 24 studies |
| - Oncology | -- | 1567 studies |
| - Tumor | -- | 19,069 studies |
| **Acceptance and Commitment Therapy** | 1 study | 319 studies |
| **Therapy** | 1 study | 147,070 studies |
| - Disease management | -- | 366 studies |
| - Therapeutic | -- | 7,375 studies |
| - Therapeutics | -- | 386 studies |
| - Treatment | -- | 111,381 studies |
| **Commitment** | 1 study | 630 studies |
| **Acceptance** | 1 study | 952 studies |

-- No studies found

* Number of studies in the search results containing the term or synonym

** Number of studies in the entire database containing in the term or synonym

| **Ebsco** | | |
| --- | --- | --- |
| **ID#** | **Query** | **Results** |
| S14 | S6 AND S10 AND S13 | 130 |
| S13 | S11 OR S12 | 3,289,735 |
| S12 | AB Randomized controlled trial OR AB Randomized OR AB Placebo OR AB Double-blind OR AB Trial | 2,988,196 |
| S11 | TI Randomized controlled trial OR TI Randomized OR TI Placebo OR TI Double-blind OR TI Trial | 999,209 |
| S10 | S7 OR S8 OR S9 | 2,595 |
| S9 | AB acceptance commitment therapy OR AB acceptance commitment therapy | 118 |
| S8 | TI acceptance commitment therapy OR TI acceptance commitment therapy | 25 |
| S7 | SU Acceptance and commitment therapy | 2,518 |
| S6 | S1 OR S2 OR S3 OR S4 OR S5 | 8,806,262 |
| S5 | AB Neoplasms, Malignant OR AB Benign Neoplasm OR AB Neoplasms, Benign OR AB Neoplasm, Benign | 10,601 |
| S4 | AB Tumor OR AB Tumors OR AB Neoplasm OR AB Neoplasia OR AB Neoplasias OR AB Cancer OR AB Cancers OR AB Malignant Neoplasm OR AB Malignancy OR AB Malignancies OR AB Malignant Neoplasms OR AB Neoplasm, Malignant | 5,690,571 |
| S3 | TI Neoplasms, Malignant OR TI Benign Neoplasm OR TI Neoplasms, Benign OR TI Neoplasm, Benign | 696 |
| S2 | TI Tumor OR TI Tumors OR TI Neoplasm OR TI Neoplasia OR TI Neoplasias OR TI Cancer OR TI Cancers OR TI Malignant Neoplasm OR TI Malignancy OR TI Malignancies OR TI Malignant Neoplasms OR TI Neoplasm, Malignant | 3,612,349 |
| S1 | SU Neoplasms | 6,512,820 |

| **Elsevier** | |
| --- | --- |
| **Query** | **Results** |
| Title, abstract, keywords: acceptance commitment cancer | 25 |

# Appendix B: Details of Risk of Bias of Included Studies.

| Authors, Year, Country | Domain 1  Randomization process | | | Domain 2  Deviations from intended interventions | | | | | | | Domain 3  Missing outcome data | | | | Domain 4  Measurement of the outcome | | | | | Domain 5  Selection of the reported result | | |
| --- | --- | --- | --- | --- | --- | --- | --- | --- | --- | --- | --- | --- | --- | --- | --- | --- | --- | --- | --- | --- | --- | --- |
|  | S  1.1 | S  1.2 | S  1.3 | S  2.1 | S  2.2 | S  2.3 | S  2.4 | S  2.5 | S  2.6 | S  2.7 | S  3.1 | S  3.2 | S  3.3 | S  3.4 | S  4.1 | S  4.2 | S  4.3 | S  4.4 | S  4.5 | S  5.1 | S  5.2 | S  5.3 |
| Burns et al. (2023), USA | PY | Y | N | PY | Y | N | NA | NA | Y | NA | PN | PY | NA | NA | N | N | N | NA | NA | Y | N | N |
| Daneshvar et al. (2020), Iran | Y | NI | N | Y | Y | N | NA | NA | Y | NA | Y | NA | NA | NA | PN | N | Y | PY | PY | Y | N | N |
| Fernández-Rodríguez et al. (2021), Spain | Y | PN | N | Y | Y | N | NA | NA | Y | NA | N | Y | NA | NA | N | N | Y | PY | PY | PY | N | N |
| Ghorbani (2021), Iran | Y | N | N | N | Y | N | NA | NA | Y | NA | Y | NA | NA | NA | N | N | PN | NA | NA | Y | N | N |
| Johns et al. (2020), USA | Y | Y | N | N | Y | N | NA | NA | Y | NA | Y | NA | NA | NA | N | PN | Y | PY | PY | Y | N | N |
| Li et al. (2022), CHINA | Y | Y | N | PY | Y | N | NA | NA | Y | NA | PN | PY | NA | NA | N | N | N | NA | NA | Y | N | N |
| Mani et al. (2019), Iran | PY | PN | N | PY | PY | N | NA | NA | PY | NA | Y | NA | NA | NA | PN | PN | Y | PY | PY | Y | N | N |
| Mosher et al. (2022), USA | PY | Y | N | PY | Y | N | NA | NA | Y | NA | PN | PY | NA | NA | N | N | N | NA | NA | Y | N | N |
| Mosher et al. (2019), USA | Y | PY | PN | PY | Y | PN | NA | NA | Y | NA | N | PN | PY | PN | PN | PN | N | NA | NA | PY | PN | PN |
| Mosher et al. (2018), USA | Y | Y | PY | Y | Y | PN | NA | NA | Y | NA | PN | Y | NA | NA | N | PN | N | NA | NA | PY | PN | PN |
| Peron et al. (2022), USA | Y | NI | N | Y | Y | PN | NA | NA | PY | NA | Y | NA | NA | NA | N | PN | Y | PY | PN | Y | N | N |
| Rost et al. (2012), USA | Y | N | NI | Y | Y | PN | NA | NA | Y | NA | N | PY | NA | NA | PN | N | Y | PY | PY | PY | PN | PN |
| Serfaty et al. (2019), UK | Y | Y | PN | PY | Y | PY | PN | NA | N | PY | N | PN | PY | PY | PN | PY | NA | NA | NA | PY | PN | PN |
| Shari et al. (2021), Malaysia | PY | PY | PN | Y | Y | N | NA | NA | PY | NA | Y | NA | NA | NA | N | PN | N | NA | NA | PY | PN | PN |
| Wells-Di et al. (2018), USA | PY | Y | N | Y | Y | N | NA | NA | Y | NA | N | PY | NA | NA | N | N | N | NA | NA | PY | PN | PN |
| Wright et al. (2023), USA | PY | N | N | Y | Y | PN | NA | NA | Y | NA | PN | PY | NA | NA | N | N | Y | PY | PN | PY | N | N |

# Appendix C: The Publication Bias Assessments Demonstrated by Funnel Plots.

**Fig. S1** The funnel plots of anxiety and depression in postintervention and follow-up

**Fig. S2** The funnel plots of AAQ-2, symptoms and QoL.

# Appendix D: Forest Plots of Secondary Outcomes.

**Fig. S3** Meta-analytic results of ACT for cancer patients on distress in post-intervention and follow-up time points.

**Fig. S4.1** Meta-analytic post-treatment result of ACT for cancer patients on other symptoms such as fatigue, insomnia and pain.

**Fig. S4.2** Meta-analytic follow-up result of ACT for cancer patients on other symptoms such as fatigue, insomnia and pain.


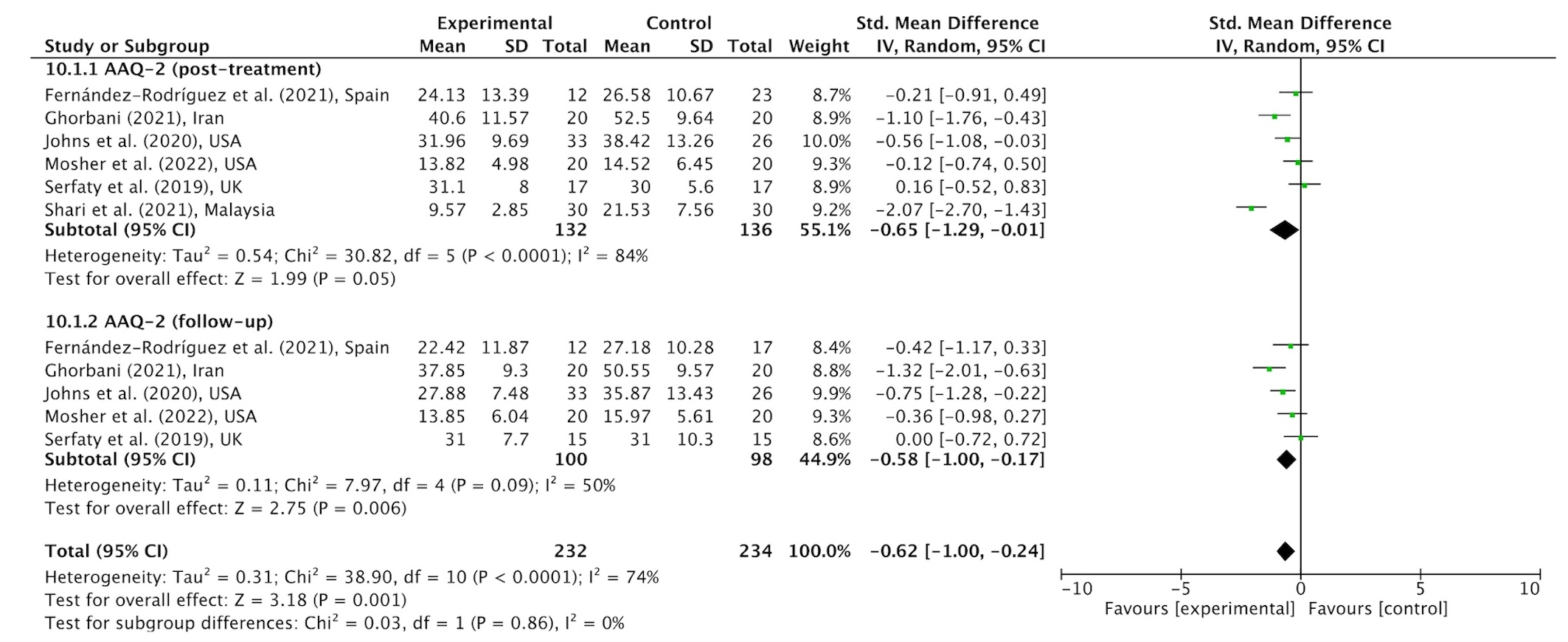


**Fig. S5** Meta-analytic results of ACT for cancer patients on AAQ-2.

**Fig. S6** Subgroup analysis of 3.4.1 according to different control conditions in post-treatment.

**Fig. S7** Subgroup analysis of 3.4.2 according to different control conditions in post-treatment.


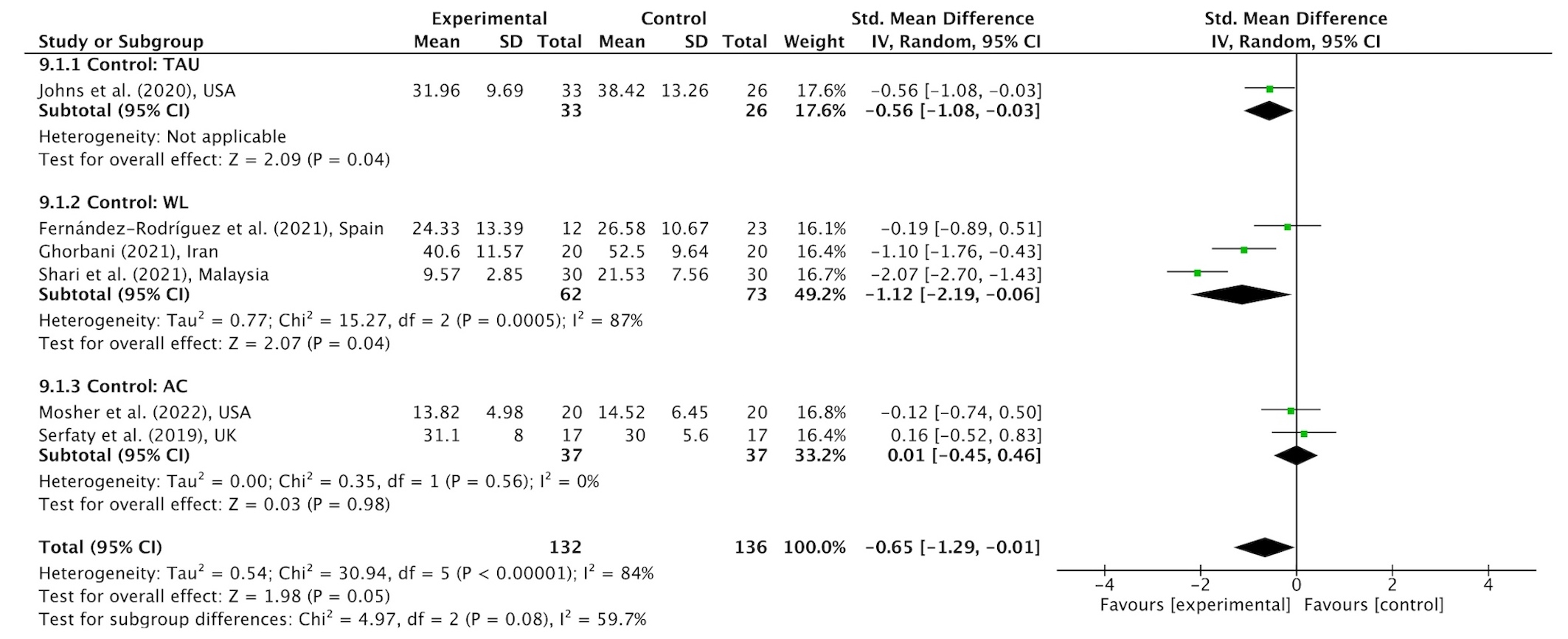


**Fig. S8.1** Subgroup analysis of post-treatment efficacy of ACT for cancer patients on AAQ-2.


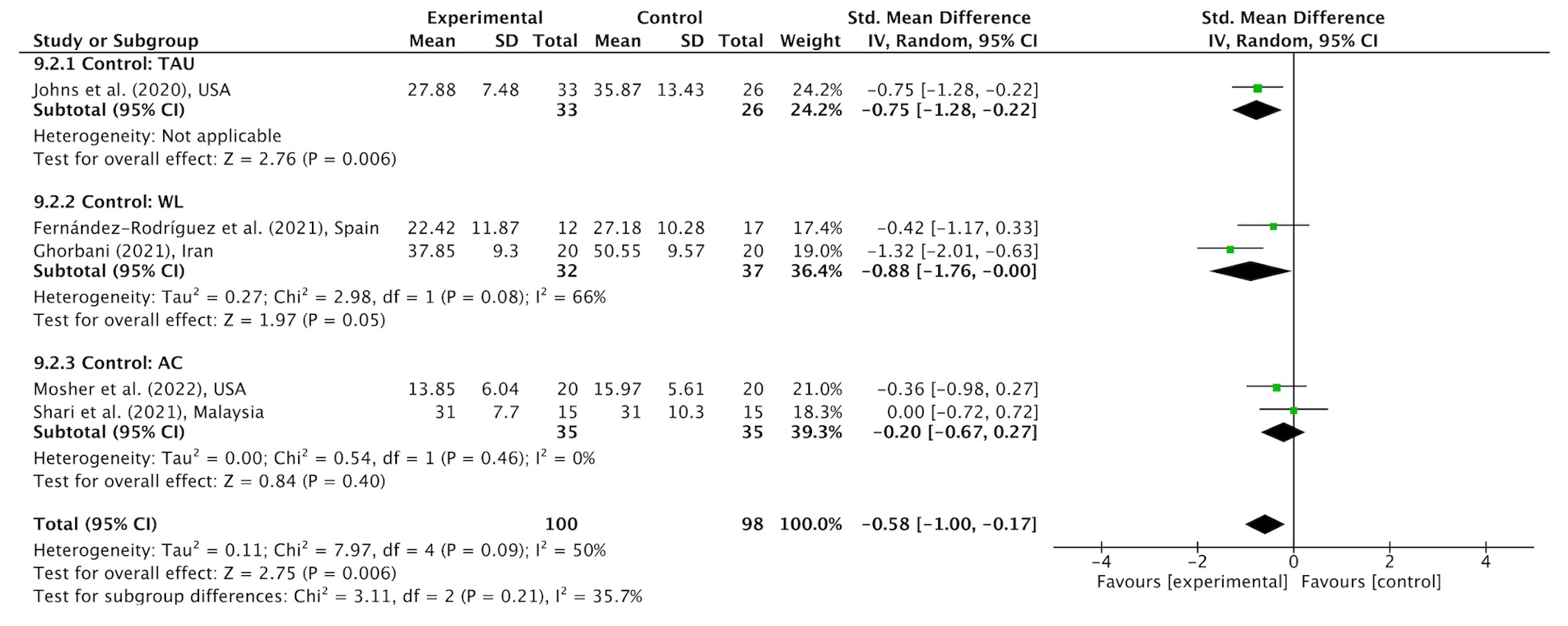


**Fig. S8.2** Subgroup analysis of follow-up efficacy of ACT for cancer patients on AAQ-2.
